# Phototoxicity of low doses of light and influence of the spectral composition on human RPE cells

**DOI:** 10.1038/s41598-024-56980-9

**Published:** 2024-03-21

**Authors:** Anaïs Françon, Kimberley Delaunay, Thara Jaworski, Cécile Lebon, Emilie Picard, Jenny Youale, Francine Behar-Cohen, Alicia Torriglia

**Affiliations:** 1 Centre de Recherche Des Cordeliers, INSERM UMRS 1138, Université Paris Cité, Sorbonne Université. Team: Physiopathology of Ocular Diseases: Therapeutic Innovations, 15, Rue de L’école de Médecine, 75006 Paris, France; 2 Assistance Publique, Hôpitaux de Paris, Hôpital Cochin, Ophtalmopole, 27, Rue du Faubourg Saint-Jacques, 75014 Paris, France; 3 Department of Ophthalmology, Hôpital Foch, 40 Rue Worth, 92150 Suresnes, France

**Keywords:** Neuroscience, Visual system, Retina

## Abstract

Light is known to induce retinal damage affecting photoreceptors and retinal pigment epithelium. For polychromatic light, the blue part of the spectrum is thought to be the only responsible for photochemical damage, leading to the establishment of a phototoxicity threshold for blue light (445 nm). For humans it corresponds to a retinal dose of 22 J/cm^2^. Recent studies on rodents and non-human primates suggested that this value is overestimated. In this study, we aim at investigating the relevance of the current phototoxicity threshold and at providing new hints on the role of the different components of the white light spectrum on phototoxicity. We use an in vitro model of human induced pluripotent stem cells (hiPSC)-derived retinal pigment epithelial (iRPE) cells and exposed them to white, blue and red lights from LED devices at doses below 22 J/cm^2^. We show that exposure to white light at a dose of 3.6 J/cm^2^ induces an alteration of the global cellular structure, DNA damage and an activation of cellular stress pathways. The exposure to blue light triggers DNA damage and the activation of autophagy, while exposure to red light modulates the inflammatory response and inhibits autophagy.

## Introduction

When light reaches the retina, visual perception is initiated. However, light is potentially toxic and will cause retinal damage under certain conditions. Solar damage to the retina was studied clinically by Duke-Elder in 1926^1^ but previous reports already pointed this phenomenon: Sir Isaac Newton suffered from retinal scotoma after observing the sun through a telescope^2^. In 1966, Noell et al.^3^ suggested that damage to the retina was also possible with low-intensity light after an extended exposure.

Retinal phototoxicity mostly affects photoreceptors and the retinal pigment epithelium (RPE). The RPE is a cellular monolayer located underneath the photoreceptor layer in the retina. It is responsible for the recycling of the photopigments to complete the visual cycle with the photoreceptors outer segments, the degradation of the photoreceptor tips through phagocytosis, and the insulation of the retina from the systemic circulation by the outer blood-retinal barrier (BRB) mediated by the tight junctions between cells. Due to its importance in photoreceptor physiology, RPE cells are essential for the survival of photoreceptors. Their damage, as seen in age-related macular degeneration for instance, can initiate the degeneration of the whole retina. RPE are sensitive to blue light radiations, a photodamage described by Ham as type II photodamage, to differentiate it from the type I that concerns photoreceptors^4,5^.

The potential of light to damage the retina has led to the definition of phototoxicity thresholds. These are used to establish safety regulations for domestic light sources. The definition of the phototoxicity thresholds for polychromatic light hypothesizes that the blue component at 445 nm is responsible for the phototoxicity. This is the so-called Blue Light Hazard (BLH). Hence, wavelengths other than blue composing the spectrum of a white light source are considered not to significantly affect its phototoxic power^6^. Interestingly, the unit used to measure light exposure is the retinal dose. The retinal dose is defined as the energy from the lighting source reaching the retina, which in turn depends on the integrated spectrum of the source, its distance from the eye, the geometry of the studied eye (see^6^) and the exposure time. So that, the exposure time is included in this parameter, measured in Joules per square centimeters (J/cm^2^). The relevance of this parameter is clearly demonstrated in the seminar work of Ham^7^ where it is shown that the same damage was found after an exposure of aphakic monkeys to a 325 nm light, either during 100 s at a retinal irradiance of 50 mW/cm^2^ or during 1,000 s at 5 mW/cm^2^, showing that the most important factor is the final retinal dose of 5 J/cm^2^.

The current phototoxicity threshold for humans has been determined using the phototoxicity curves published by Van Norren and Gorgels^6^ and originally obtained from primates. For blue light at 445 nm, the threshold is a retinal dose of 22 J/cm^2^. By using a safety factor of 10, the NF EN 62,471 safety regulation derived from this threshold sets that a light source is totally harmless if it delivers a BLH-weighted retinal dose below 2.2 J/cm^2^ in 10,000 s when placed at 20 cm from the eye.

It is worth noting that phototoxicity studies using primates are sparse and rodent models are more frequently used in such studies. In the last years, several studies brought to light an overestimation of the current phototoxicity threshold for rodents and primates^8,9^. This was reported by Hunter et al.^8^ that found that the phototoxicity thresholds for primates were overestimated by a factor of 20, and Jaadane et al.^9^ that found in rodents a similar value. This raises questions about the accuracy of the current phototoxicity threshold for humans.

In this paper, we aim at giving some information regarding the relevance of the current phototoxicity threshold for humans. We investigated this concern by exposing human-induced pluripotent stem cells (hiPSC)-derived retinal pigment epithelial (iRPE) cells to doses of light below 22 J/cm^2^ followed by an evaluation of the impact on the integrity of the iRPE cells. We also focused on the importance of the light spectrum on phototoxicity by highlighting the different reactions of the iRPE cells after exposure to different wavelengths. We used blue wavelengths because they are the most energetic and involved in phototoxicity, and red wavelengths because, contrary to the blue ones, they are the less energetic of the visual spectrum and were shown to have protective effects in many systems (a phenomenon known as photobiomodulation)^10,11^.

## Results

### Light impacts the overall cellular structure

**Figure 1:**
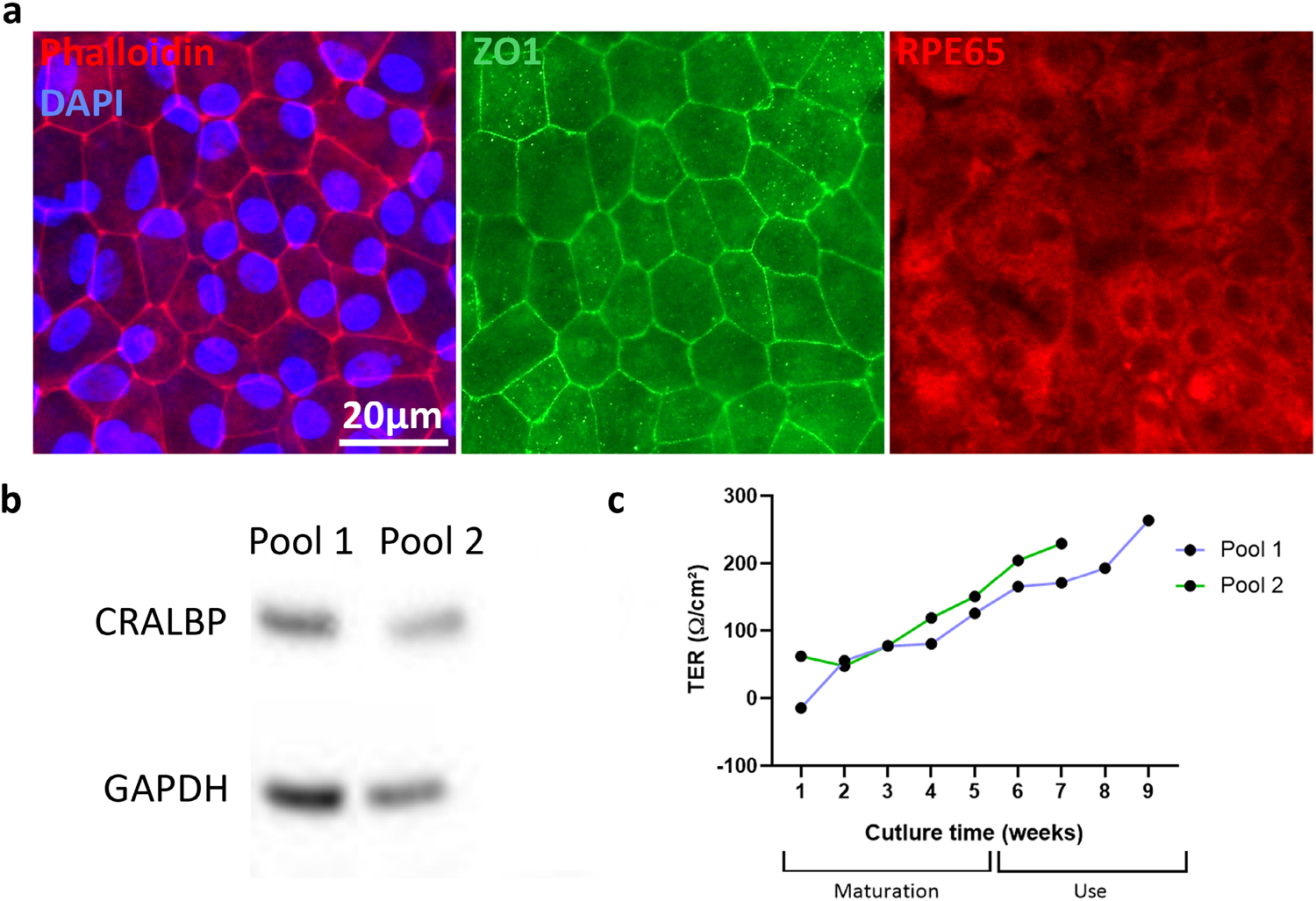
Characterization of the iRPE cells. (**a**) Immunostaining showing major key characteristics of RPE cells. Left panel: actin cytoskeleton (phalloidin-FITC, red) with nuclei (DAPI, blue), middle panel: tight junctions (anti-ZO1, green), and right panel: (anti-RPE65, red). (**b**) Western blot showing the expression of CRALBP in the two pools of iRPE cells used in this study. (**c**) Trans-epithelial resistance (TER) measurement during the culture time of the iRPE cells in transwells for the two pools of used cells.

First, we confirmed the RPE phenotype of the obtained iRPE cells. The actin cytoskeleton organization in the cells, as shown by the phalloidin staining, was characteristic of an epithelial monolayer with joining cells surrounded by an actin belt and expressing the tight junction protein zonula occludens 1 (ZO1) at their membrane (Fig. 1a). Moreover, both pools of iRPE cells used expressed key RPE proteins such as retinal pigment epithelium-specific 65 kDa (RPE65), visible by immunostaining, and cellular retinaldehyde-binding protein (CRALBP), visible by western blot (Fig. 1b). Finally, the trans-epithelial resistance (TER) of the iRPE cells gradually increased to reach levels above 100 Ω/cm^2^ at the end of the maturation period, as previously reported for these cells^12^ (Fig. 1c).

**Figure 2:**
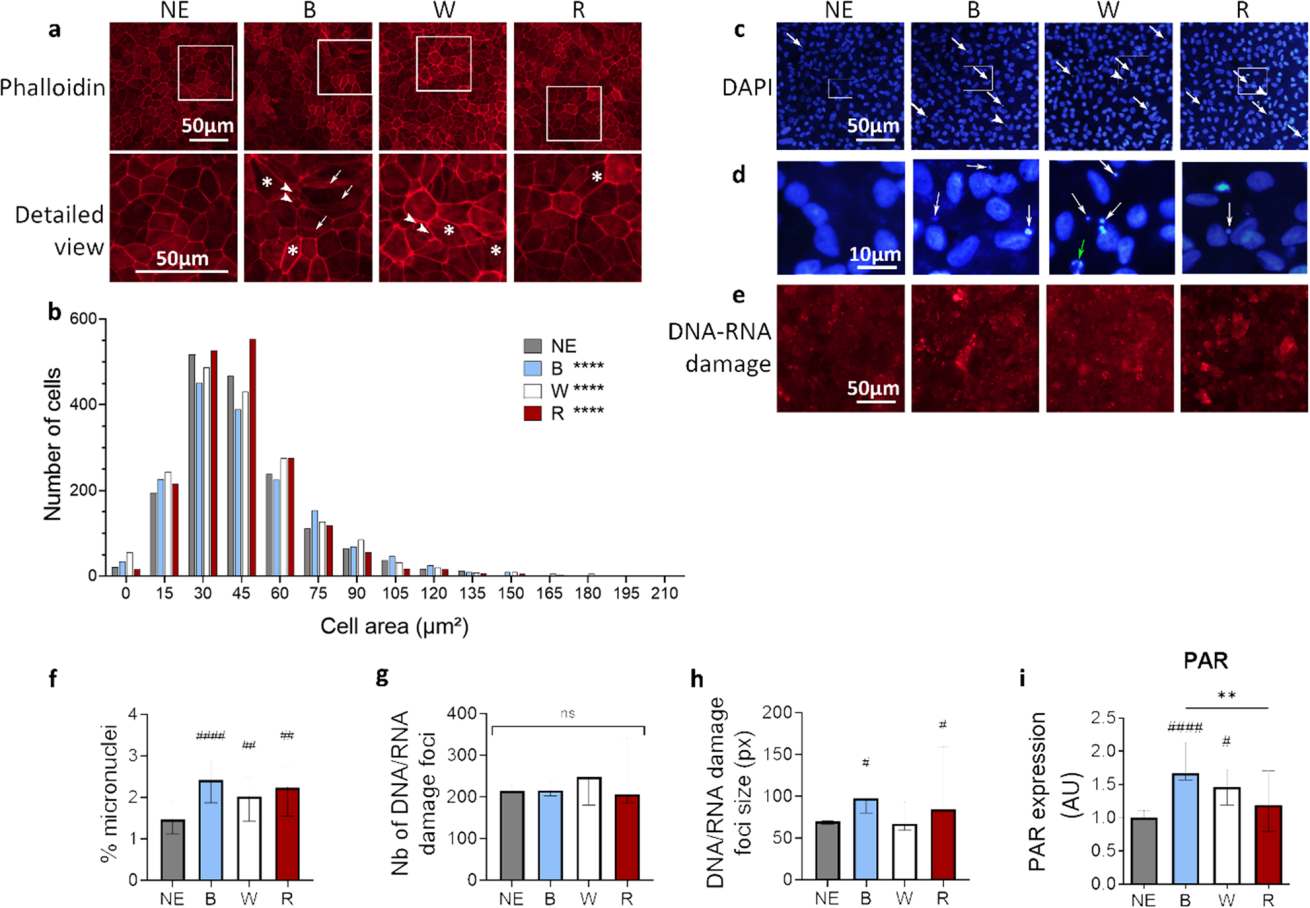
Impact of light exposure on RPE cell structure and DNA. (**a**) Actin cytoskeleton staining using phalloidin of iRPE cells non-exposed (NE) or exposed to blue (B), white (W) or red (R) light. Arrowheads show small cells. Asterisks show elongated cells. Arrows show stress fibers. (**b**) Distribution of the cell area of iRPE cells. The cells were classified depending on their area using a bin of 15 µm^2^. The x axis corresponds to the bin center. The first bin (0) corresponds thus to cells ≤ 7.5 µm^2^. The statistical analysis was performed using a Chi-square test comparing the distribution of the exposed conditions (observed) with the distribution of the non-exposed cells (expected). n = 2 wells per condition (n ≥ 1648 cells per condition). ****p < 0.0001 compared to NE. (**c**) DAPI staining of iRPE cells NE or exposed to B, W or R light. White arrows show micronuclei. Arrowheads show pyknotic nuclei. (**d**) High magnifications of panel b. White Arrow show micronuclei, green arrow an apoptotic cell. (**e**) Anti-DNA/RNA damage immunolabeling, targeting 8-hydroxy-guanosine, on iRPE cells NE or exposed to B, W or R light. (**f**) Quantification of the proportion of micronuclei observed in transwells of iRPE cells NE or exposed to B, W or R light. H(4) = 16.74, n = 24 wells per condition. (**g**) Counting of the number of DNA/RNA damage foci per image in transwells of iRPE cells NE or exposed to B, W or R light. H(4) = 0.7721, n = 4 wells per condition. (**h**) Measurement of the size of the DNA/RNA damage foci in transwells of iRPE cells NE or exposed to B, W or R light. H(4) = 7.676, n = 4 wells per condition. (**i**) Quantification by western blot of the quantity of Poly-ADP Ribose polymer (PAR) in iRPE cells exposed to light. H(4) = 21.22, n = 12 wells per condition. (**e**–**i**) Graphs represent the median with the interquartile range. Statistical analysis was performed using the Kruskal–Wallis test followed by Dunn’s post-test. ns: non-significant between all groups, #: compared to NE, #*p* < 0.05, ##*p* < 0.01, ###*p* < 0.001, ####*p* < 0.0001, **p < 0.01.

The impact of an exposure to light emitting diode (LED) light of different wavelengths was first observed on the global structure of the cell layer through staining of the actin cytoskeleton. iRPE cells exposed to blue and white lights appeared less homogeneous in size and shape than non-exposed cells (Fig. 2a). Numerous very small iRPE cells (arrowheads, Fig. 2a) were mixed with extremely elongated cells (asterisks, Fig. 2a) after exposure to blue and white lights. Stress fibers appeared in the actin cytoskeleton (arrows Fig. 2a) after blue light exposure. In contrast, iRPE cells exposed to red light looked similar to the non-exposed cells. A morphological analysis of the cells confirmed these observations (Fig. 2b). It showed that the distribution of the cell area varied significantly between the non-exposed and the cells exposed to light. The red-exposed cells were enriched in middle-size cells, while the blue- and white-exposed cells were enriched in small and big cells.

Observation of nuclei using DAPI staining highlighted the presence of a few apoptotic nuclei (arrowheads, Fig. 2c,d) and a significant increase in the number of micronuclei (arrows, Fig. 2c,d, quantification Fig. 2f) after exposure to the three light conditions. As these micronuclei could be a sign of nuclear fragmentation following DNA damage, a labeling of DNA and RNA damage was then performed. Whilst the number of damage foci did not increase in light-exposed cells as compared to the non-exposed cells (Fig. 2e,g), the size of the damage foci significantly increased following exposure to blue and red lights (Fig. 2h). Finally, the presence of poly (ADP-ribose) (PAR) polymer was studied by western blot, as PAR is involved in the maintenance of DNA integrity and as the PARylation of the histones helps recruiting repair proteins in the context of DNA breaks. Exposure to both blue and white lights led to the accumulation of PAR in the iRPE cells compared to non-exposed cells. But exposure to red light did not increase the amount of PAR (Fig. 2i). These results indicate that different wavelengths induce different DNA damage and repair mechanisms.

### Light induces an inflammatory stress on the iRPE

**Figure 3:**
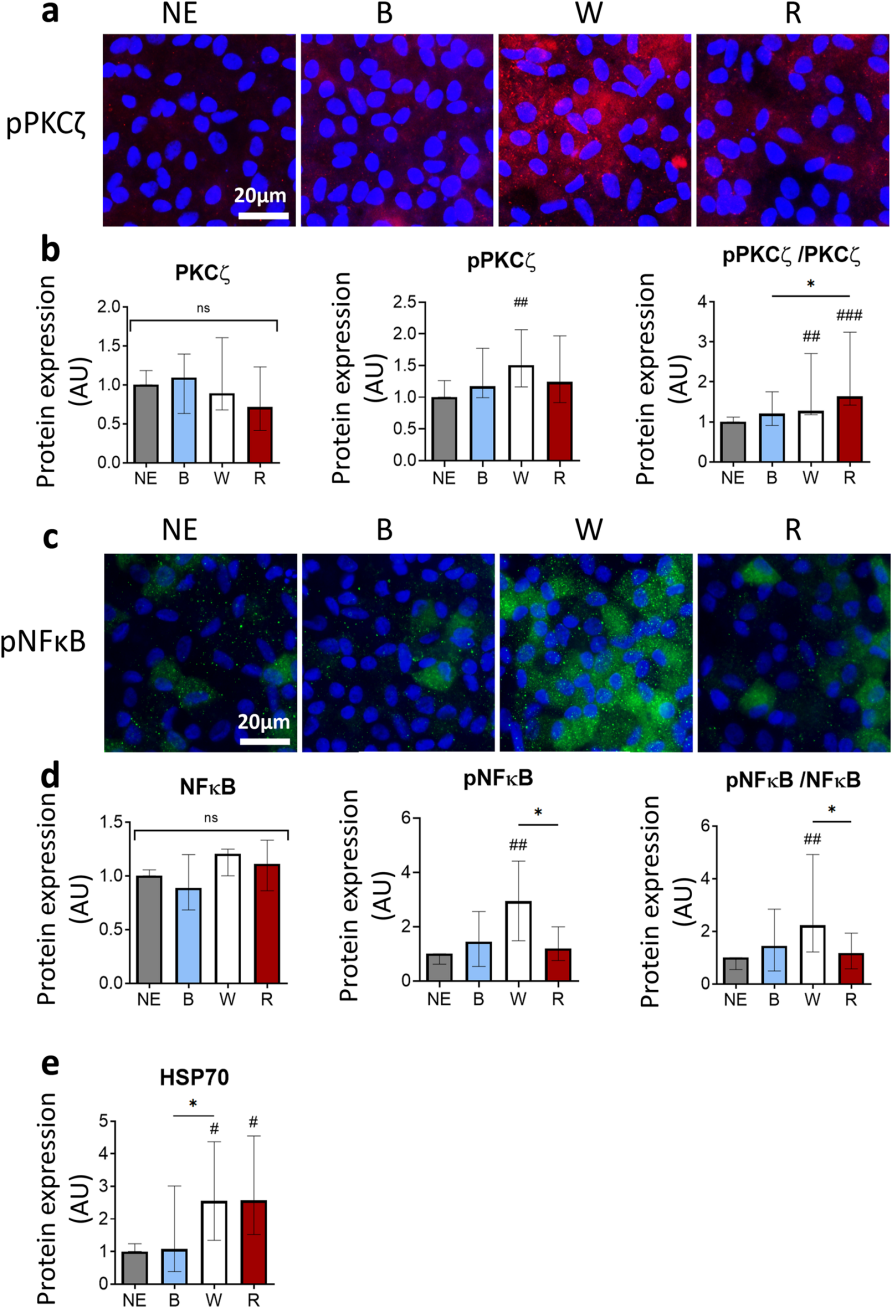
Impact of light exposure of RPE cells on stress and inflammatory pathways. (**a)** Anti-pPKCζ (Thr410) and DAPI immunostaining of iRPE cells non-exposed (NE) or exposed to blue (B), white (W) or red (R) light. (**b**) Quantification by western blot of the PKCζ protein, pPKCζ (Thr410), and of the ratio pPKCζ/PKCζ in iRPE cells exposed to light. PKCζ: H(4) = 3.928, n = 12 wells per condition. pPKCζ: H(4) = 6.713, n = 12 wells per condition. pPKCζ/PKCζ: H(4) = 15.42, n = 12 wells per condition. (**c**) Anti-pNFκB (Ser311) and DAPI immunostaining of iRPE cells NE or exposed to B, W or R light. (**d**) Quantification by western blot of the NFκB protein, pNFκB (Ser311), and of the ratio pNFκB/NFκB in iRPE cells exposed to light. NFκB: H(4) = 4.066, n = 12 wells per condition. pNFκB: H(4) = 10.99, n = 12 wells per condition. pNFκB/NFκB: H(4) = 8.917, n = 12 wells per condition. (**e**) Quantification by western blot of the quantity of HSP70 in iRPE cells exposed to light. HSP70: H(4) = 10.05, n = 12 wells per condition. Graphs represent the median with the interquartile range. Statistical analysis was performed using the Kruskal–Wallis test followed by Dunn’s post-test. ns: non-significant between all groups, #: compared to NE, #*p* < 0.05, ##*p* < 0.01, ###*p* < 0.001, *p < 0.05.

Previous studies on rat highlighted that exposure to white light from LED devices induced the activation of the Nuclear Factor-kappa B (NFκB) pathway via the Protein Kinase C zeta (PKCζ) in the retina^9,13^. Activation of PKCζ by phosphorylation of threonine 410 induces phosphorylation and activation of NFκB on serine 311. Whilst immunostaining did not show increased pPKCζ in the iRPE cells after light exposure (Fig. 3a), quantification by western blotting highlighted an increased phosphorylation of PKCζ after white and red lights exposure (Fig. 3b), whereas the global amount of PKCζ remained stable for all conditions. Subsequently, the number of iRPE cells highly stained with anti-phosphorylated NFκB increased after exposure to white light (Fig. 3c). This observation was confirmed by western blot quantification. While the global amount of NFκB protein was stable across the different conditions, the proportion of phosphorylated NFκB protein was higher in white light-exposed cells compared to non-exposed cells and red light-exposed cells (Fig. 3d). Thus, exposure to white and red lights, but not blue light, activated PKCζ. However, only white light exposure showed a significant increase in NFκB activation downstream of PKCζ.

Another target of activated PKCζ is the Heat Shock Protein 70 (HSP70), as pPKCζ is involved in the recruitment of HSP70 to lipid rafts to induce an inflammatory response. Quantification by western blot showed an increase of HSP70 in the cells exposed to white and red lights compared to the non-exposed cells (Fig. 3e). The amount of HSP70 was also significantly higher in cells exposed to white light compared to cells exposed to blue light. It is to note that HSP70 is often used as a marker of the response to heat shock. In this study, the temperature inside the incubator had been monitored during the exposure to LED light and showed no raise of temperature.

### Light modulates the autophagic pathway

**Figure 4:**
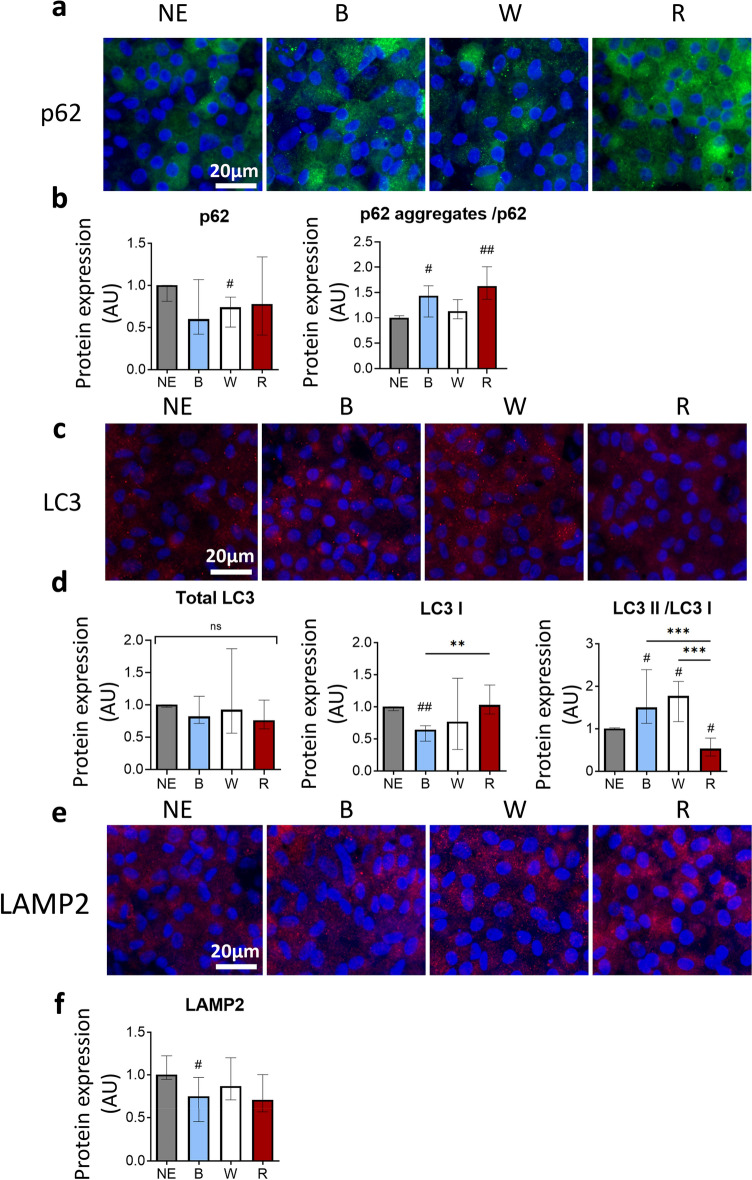
Impact of light exposure of RPE cells on autophagy. (**a**) Anti-p62 and DAPI immunostaining of iRPE cells non-exposed (NE) or exposed to blue (B), white (W) or red (R) light. (**b**) Quantification by western blot of the expression of p62 and of the ratio of p62 aggregates/p62 in iRPE cells exposed to light. p62: H(4) = 6.316, n ≥ 11 wells per condition. p62 aggregates/p62: H(4) = 10.73, n ≥ 11 wells per condition. (**c**) Anti-LC3 and DAPI immunostaining of iRPE cells NE or exposed to B, W or R light. (**d**) Quantification by western blot of the total amount of LC3 (LC3 I + LC3 II), the LC3 I form, and the ratio LC3 II/LC3 I form in iRPE cells exposed to light. Total LC3: H(4) = 4.77, n = 12 wells per condition. LC3 I: H(4) = 10.45, n = 12 wells per condition. LC3 II/LC3 I: H(4) = 28.18, n = 12 wells per condition. (**e**) Anti-LAMP2 and DAPI immunostaining of iRPE cells NE or exposed to B, W or R light. (**f**) Quantification by western blot of LAMP2 expression in iRPE cells exposed to light. H(4) = 6.329, n = 12 wells per condition. Graphs represent the median with the interquartile range. Statistical analysis was performed using the Kruskal–Wallis test followed by Dunn’s post-test. ns: non-significant between all groups, #: compared to NE, #*p* < 0.05, ##*p* < 0.01, **p < 0.01, ***p < 0.001.

The p62 protein, also called Sequestosome 1 (SQSTM1), aggregates with polyubiquitylated proteins to drive them to the autophagosomes for degradation. Immunostaining and western blot quantification of p62 highlighted an increase of the p62 aggregates in iRPE cells exposed to blue and red lights (Fig. 4a,b). In contrast, cells exposed to white light displayed a reduced amount of the p62 protein, suggesting an involvement of autophagy in response to light. The Light Chain 3 (LC3), a marker of autophagosome formation, is a cytosolic protein (LC3 I) converted into LC3 II to be integrated to the autophagosome. No major change in LC3 staining was visible on cells exposed to different lighting conditions (Fig. 4c) and its total amount remained stable (Fig. 4d). However the quantity of LC3 I decreased after the exposure to blue light leading to a LC3 II/LC3 I ratio significantly enhanced when cells were exposed to blue or white lights. By contrast, the LC3 II/ LC3 I ratio was sharply reduced by the exposure to red light. To complete the autophagic pathway, autophagosomes fuse with lysosomes using the lysosome-associated membrane protein 2 (LAMP2). Staining of LAMP2 showed that exposed cells were similar to non-exposed cells (Fig. 4e). Quantification of LAMP2 by western blot did not confirm this but revealed a significant reduction of LAMP2 in cells exposed to blue light (Fig. 4f).

## Discussion

This study shows that the exposure of human RPE cells to doses of light below the currently admitted phototoxicity threshold for blue light (22 J/cm^2^) induces an alteration of the global structure of the cell layer, DNA damage, activation of cellular stress and modulation of autophagy. The impact of the light is wavelength-dependent, blue and red lights often displaying opposite effects^14,15^.

In this work we exposed hiPSC-derived RPE cells to a single dose^7^ of white light of 3.6 J/cm^2^, corresponding to 0.185 J/cm^2^ of blue light and 0.276 J/cm^2^ of red light according to the energy distribution of its spectrum. Table 1 shows an overview of the obtained results.

**Table 1:** Overview of the results obtained in this study.

	Blue	White	Red
Cell structure	Modified	Modified	Ø
Micronuclei	+	+	+
DNA/RNA foci #	Ø	Ø	Ø
DNA/RNA foci size	+	Ø	+
PAR	+	+	Ø
HSP70	Ø	+	+
pPKCζ/PKCζ	Ø	+	+
pNFκB/NFκB	Ø	+	Ø
p62 aggregates	+	Ø	+
LC3 II/LC3 I	+	+	−
LAMP2	−	Ø	Ø

Although the exposure limits for a total dose of polychromatic light are not defined, the energy threshold for human’s retina in blue light, which is thought to be the only responsible of retinal phototoxicity, is 22 J/cm^2^. The exposure corresponding to blue light represents here 0.185 J/cm^2^. So, although some of our results seem mild, we are far below the toxic dose, in a range where we are not supposed to bring out any effect. However, several changes are highlighted. First, the cell shape becomes less homogeneous and stress fibers appear. This is seen in white and blue light exposure while red light has no effect, suggesting that blue light is responsible for this morphological modification, as expected^16^. It is to note that similar results were found at these doses in the RPE of light-exposed rats^9^.

Exposure of human RPE cells derived from hiPSC induces DNA damage as indicated by the increased amount of micronuclei together with a significant increase of polyADP ribose polymer (PAR), indicating the presence of DNA damage and the activation of the signal for DNA repair^17^. When investigating DNA damage, we find that all exposure conditions lead to micronuclei increase but only white and blue lights activate the synthesis of PAR, suggesting that blue light is responsible for most of the DNA damage. The absence of PAR increase together with the increase of HSP70 (involved in DNA repair of single-strand breaks^18^) and the increase of micronuclei in cells exposed to red light as compared with controls is intriguing. These results could be in favor of a modulation of DNA damage sensing and/or repair mechanisms by red wavelengths. Actually, photobiomodulation studies have shown that red light exposure enhances the activity of GADD45A (growth arrest and DNA damage inducible, alpha)^19^, and alters the expression of ERCC1/2 proteins (excision repair cross-complementing group 1/2)^20^. This issue is currently under investigation.

Exposure to white light also results in the activation of the PKCζ/NFκB pathway as seen by the increased phosphorylation of both PKCζ and NFκB. These results, together with the increase of HSP70, are in accordance with those obtained in rodents^13^ and suggest the activation of a cellular inflammatory response^21^. Note that over-activation of PKCζ in RPE is involved in the loss of the RPE barrier integrity^13,22–25^. However, blue light exposure does not activate this pathway, suggesting that the blue part of the spectrum is not responsible for this effect. This in in favor of a role of the other parts of the white light spectrum, such as green wavelengths.

Interestingly, exposure to red light induces an activation of PKCζ without NFκB phosphorylation and an increase in p62 aggregates. This suggests an inhibition of the PKCζ/NFκB pathway downstream of PKCζ, perhaps because of the aggregation of p62, a protein that, in its native state, promotes PKCζ recruitment to the NFκB complex^26^. Moreover, the increase in the expression of HSP70 has been shown to reduce cellular inflammation by inhibiting the NFκB pathway^27^. So that, our results seem to be in line with an inhibition of cellular inflammatory pathways by red light, already shown in different models of photobiomodulation^28,29^.

Concerning the autophagic pathway, which is extremely important for RPE cells^30^, iRPE cells exposed to white light display a higher LC3 II/ LC3 I ratio, with no change in LAMP2 or p62 aggregates. This is in favor of the activation of autophagy that appears sufficient to prevent the accumulation of these aggregates. Exposure to blue light also induces an increase of the LC3 II/ LC3 I ratio, but this goes along with the decrease of LAMP2, indicating an activation of autophagy that is unable to completely prevent the aggregation of p62, as aggregates are increased in this condition. This suggests that blue light induces an overload of the autophagic pathway. Red light exposure has a contrasted effect. The decrease in LC3 II/ LC3 I ratio associated with the increase in p62 aggregates suggest that autophagy is not activated, or even inhibited. This is also coherent with the increase of the HSP70 which has the ability to inhibit the autophagy through the Akt/MTOR signaling pathway^31^. These results differ from those of Khan et al.^32^ who showed that exposure to near-infrared laser induces the activation of autophagy, but this discrepancy may depend on the illumination wavelength (810 nm for Khan against 630 in this work).

Activation of autophagy is mostly described as a cell protecting mechanism^30,33–36^. However, an excessive activation of this mechanism has been found in rodent models of retinal degeneration^37^, but has also been shown to mediate cell death in cultured RPE cells^38^. Interestingly, Mallory bodies, found in protein aggregation diseases, are formed when the refolding of proteins dependent of HSP70, and the protein degradation involving p62 are overwhelmed^39^. Hence, the simultaneous increase of HSP70 and p62 aggregates induced by exposure to red light may be seen as an attempt of the cell to aggregate the damaged proteins while waiting for the autophagic and chaperone mechanisms to unblock.

In the whole, both blue and white lights seem to activate autophagy while red light does not, showing that autophagy is modulated in a wavelength-dependent manner. A study using activators and inhibitors of autophagy, like rapamycin and 3-Methyladenine, remains to be performed to better understand these data.

As previously said, the blue component of white light is thought to be responsible for its toxicity^4–6^. However, we found significant differences between cells exposed to blue and white light, suggesting that, although blue light plays an important role in white light toxicity, it is not solely responsible for its harmful effects and that green wavelengths could also play a role in the white light toxicity. By contrast, the red wavelengths seem to be involved in the modulation of the inflammatory response, being protective for the cells^40,41^.

Taken together, our results also show that a low dose of white light (3.6 J/cm^2^), that is much below the accepted phototoxicity threshold of 22 J/cm^2^, negatively affects human iRPE cells, supporting the conclusions of other authors claiming that the toxic threshold for light toxicity is overestimated^8,13^.

We are aware that phototoxicity thresholds for the human eye cannot be calculated from this cellular model and it is not the goal of this study. However, this model appears closer to the physiological reality of the human eye than phototoxicity studies performed on non-differentiated ARPE-19 cells for which the phenotype and gene expression greatly differ from the ones of native RPE cells^42^. Moreover, we are working here with isolated cells and thus during light stress, the cells are not supporting the phagocytosis of the oxidized tips of photoreceptors that add a supplementary stress. With these limitations in mind, this study strikes the importance of the spectral composition of the light on the RPE homeostasis. The blue component not being the solely responsible for damage, a well-balanced proportion of blue and red wavelengths is crucial to reduce phototoxicity risks, even at very low energy. It is to note that light from incandescent bulbs, the most used type of device in the last century, was rich in red wavelengths. These devices are now replaced by light emitting diodes (LED), rich in blue wavelengths and extremely poor in red wavelengths. Such imbalanced light spectrum being extensively used in our daily life, the long-term consequences on retinal health should raise concern and be prospectively studied. In addition, despite the energy delivered by artificial light being lower than the solar light, this exposure is added to the natural exposure every day of our lives.

## Material and methods

### Cell culture and differentiation

A human-induced pluripotent stem cells (hiPSC) cell line, obtained from fibroblasts of a healthy donor with no known retinal disease was provided by Dr. David M. Gamm^43^ (Department of Ophthalmology and Visual Sciences, University of Wisconsin-Madison, Madison, United States, MTA 03/03/2020). The hiPSC were expanded and differentiated in our laboratory into hiPSC-derived RPE (iRPE) cells using a differentiation protocol described previously^43^ with some modifications^12,44^.

Subconfluent hiPSC cultured on hESC-Qualified Matrigel® Matrix (Corning, Boulogne-Billancourt, France) with mTeSR^TM^1 medium (Stemcell Technologies, Germany) were detached with Type IV Collagenase (Thermo scientific, Illkirch, France) to transfer 50–300 µm diameter hiPSC colonies into flasks of 25 cm^2^ (TPP Techno Plastic Products AG, Switzerland) containing embryoid body medium (DMEM/F-12, HEPES medium (Thermo scientific); 1% N-2 Supplement [100X] (Thermo scientific); 1% B-27™ Supplement [50X], serum free (Thermo scientific); 1% L-Glutamine [200 mM] (Thermo scientific); 0.1 mM 2-Mercaptoethanol (Merck, Darmstadt, Germany); 5 μM RHO/ROCK pathway inhibitor Y-27632 (Stemcell Technologies)), to form three-dimensional embryoid body-like (EB) aggregates. From here on, the different EB were differentiated independently. On day 1, EB were plated on cell culture dishes (P60) (TPP Techno Plastic Products AG) coated with Growth Factor Reduced (GFR) Basement Membrane Matrigel® Matrix (Corning) and cultured for 10 days in neural induction medium (NIM) (DMEM/F-12, HEPES medium (Thermo scientific); 1% N-2 Supplement [100X] (Thermo scientific); 1% L-Glutamine [200 mM] (Thermo scientific); 1% MEM Non-Essential Amino Acids Solution [100X] (Thermo scientific); Heparin, Sodium Salt, Porcine Intestinal Mucosa, [100KU] (MERCK)). On day 10, NIM was replaced by Retinal Differentiation Medium with vitamin A (RDMwA) (DMEM, high glucose, GlutaMAX™ Supplement, HEPES medium (Thermo scientific)/Ham's F-12 Nutrient Mix medium (Thermo scientific) [3:1]; 2% B-27™ Supplement [50X], serum free (Thermo scientific)) for the next three consecutive days. On day 13, RDMwA was switched to retinal differentiation medium without vitamin A (RDMw/oA) (DMEM, high glucose, GlutaMAX™ Supplement, HEPES medium (Thermo scientific)/Ham's F-12 Nutrient Mix medium (Thermo scientific) [3:1]; 2% B-27™ Supplement [50X], minus vitamin A (Thermo scientific)). When satisfactory level of pigmentation was reached (day 30–40), iRPE pigmented foci were manually microdissected and collected from differentiating hiPSC cultures under a microscope using a surgical blade (Paramount, New Delhi, India), seeded on Basement Membrane Matrigel® Matrix-coated P60 and grown for an additional 30 days in RDMw/oA until deeply pigmented monolayers re-formed. On day 60–70, mature iRPE cells (10^5^–2 × 10^5^) were resuspended in RDMw/oA containing 5 μM of RHO/ROCK pathway inhibitor Y-27632 and then reseeded on GFR Basement Membrane Matrigel® Matrix-coated 24-well plates (Thermo scientific) for further expansion and maturation. At this stage, iRPE cells were considered to be at passage 1 (P1) and cultured in the serum- and antibiotic-free RDMw/oA.

At passage 3, iRPE cells were seeded in Transwells (0.4 μm pore polyester membrane insert, Corning, CLS3450 and CLS3460) coated with red phenol-free matrix (Corning, Matrigel®) in RDMw/oA. Experiments were performed from day 42 of P3. One week before light exposure, iRPE cells medium was replaced by red phenol-free DMEM (high glucose, HEPES, Thermo scientific) with 10% Fetal Bovine Serum (FBS) charcoal stripped for trans-epithelial resistance stability. The same medium was used during light exposure.

All cultures were maintained in an incubator at 37 °C, 5% CO_2_. Pigmented iRPE were previously characterized according to protocol described in Canonica et al.^12^. The trans-epithelial resistance was > 100 Ω/cm^2^ after 6 weeks and heavily pigmented cells formed a monolayer (see results). Two independent pools of cells were used for this study.

### Trans-epithelial resistance measurement

The trans-epithelial resistance (TER, in Ω/cm^2^) was measured using the EVOM2 Epithelial Voltohmmeter and an STX2 electrode (World Precision Instruments, Sarasota, Florida). Each value corresponds to the average of several independent wells and was corrected for background resistance using a blank well with culture medium.

### Light exposure

**Figure 5:**
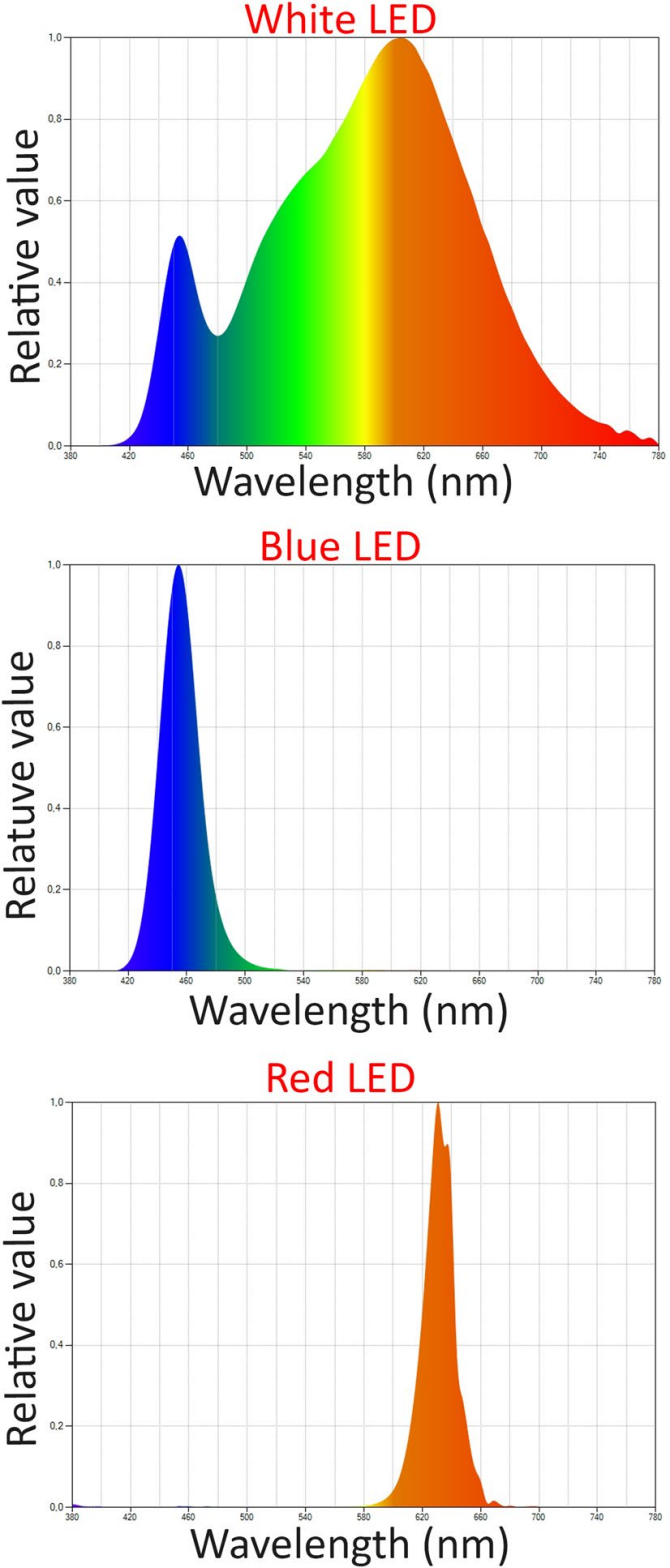
Emission spectra of the LED lights used in this study.

Cells were exposed to blue (455 nm, Joyland, D50SWD-B), white (3,300 K, Lepro4100067-WW-EU-NF-a) or red (630 nm, Qasim1xQA-SL0001-EU) LED. The specificities of each light source (CT, peak of emission, integrated irradiance, etc.) were measured using a spectroradiometer (Kanonica Minolta CL70 F CR I). Their spectra are shown in Fig. 5. Cells were exposed to white LED during 10,000 s to match the current definition of the limit exposure value. This exposure time corresponds to a dose of 3.6 J/cm^2^ for the white LED (calculated as the energy of the integrated spectrum multiplied by the exposure time). Exposure time for blue and red LED were deduced from the settings for white LED to match their proportion in the white light spectrum. As a result, iRPE cells were exposed to blue LED for 1,100 s and to red LED for 9,000 s, resulting in a dose of 0.185 J/cm^2^ for blue light and 0.276 J/cm^2^ for red light. Cells were left in the dark for 24 h following the beginning of the light exposure.

### Immunostaining

iRPE cells cultured in transwells for immunostaining were fixed using 4% paraformaldehyde for 15 min and then washed with Phosphate-Buffered Saline (PBS). The transwell’s membranes were cut and permeabilized with 0.3% Triton X-100 for 15 min at room temperature. Membranes were incubated with the primary antibodies (1:100 in PBS with 1% Bovine Serum Albumin (BSA)) for one hour (Table 2), followed by the incubation with the secondary tagged antibodies (1:200 in PBS with 1% BSA) for one hour. Then, they were incubated with DAPI (4’, 6 diamidino-2-phenylindole) (1:1000 in PBS) for 5 min, followed, for some membranes, by an incubation with AlexaFluor 594-phalloidin (Santa Cruz, Heidelberg, Germany, 1:1000 in PBS with 1% BSA) for 5 min. Finally, cells on transwell’s membranes were mounted between a slide and a coverslip with Dako Fluorescent Mounting Medium (ref S3023).

**Table 2:** List of the antibodies used in this study.

Antibody	Reference	Application
DNA-RNA damage	Abcam ab62623	IF
Poly(ADP-ribose) (PAR)	Enzo ALX-210-890A	WB
LC3B	Cell Signaling #2775	IF/WB
LAMP2	Milliport MABC40	IF/WB
p62/SQSTM1	Sigma P0067	IF/WB
pPKCζ (Thr410)	Santa Cruz sc-12894-R	IF/WB
PKCζ	Santa Cruz sc-216	WB
pNFκB (Ser311)	Santa Cruz sc-33039	IF/WB
NFκB	Santa Cruz sc-7151	WB
HSP70	BD Biosciences 610,607	WB
GAPDH	Life technologies AM4300	WB

IF: immunofluorescence. WB: western blot.

Images were taken using a fluorescent microscope (Olympus BX51, oil immersion 40 × objective). The quantification of the proportion of micronuclei was performed on the images of 24 wells per conditions stained with DAPI using ImageJ software. We considered a micronuclei as having a diameter of about sevenfold smaller than a nucleus (this means that its volume is about 300 fold smaller than the volume of the nucleus). The number of micronuclei was divided by the total number of counted nuclei (n > 50,000 nuclei per condition). Wells stained with anti-DNA/RNA damage antibodies were used to analyze the number and the size of the damage foci. We used the Analyze particles tool from ImageJ to obtain the number and the average size of the damage foci per image (n ≥ 5 images per well, 4 wells per condition).

### Protein extraction and western blot analysis

Proteins were extracted using 200 µL of M-PER Mammalian Protein Extraction Reagent (Thermo Scientific) per transwell, for 15 min in ice. The solution was then centrifuged for 5 min, 15,000 g at 4 °C. The supernatant was collected and stored at − 20 °C. Protein concentration was determined using the bicinchoninic acid (BCA)™ Protein Assay Kit (Thermo Scientific) following the manufacturer’s instructions.

Protein samples were mixed with Laemmli sample buffer (10% Glycerol, 2% SDS, 100 mM Dithiothreitol, 62.5 mM Tris, Bromophenol blue) for western blot analysis. 20 µg of proteins were loaded per well of a Bolt™ 4–12% Bis–Tris Plus gel (Thermo Scientific, ref NW04125BOX). Then, proteins were transferred on a nitrocellulose membrane (Protan®, Whatman®, GE Healthcare, Versailles, France). After one hour of saturation with 5% dry skimmed milk in PBS at room temperature, membranes were incubated for 2 h with the primary antibody (1:1000 in PBS with 0.5% milk and 0.1% Tween, Table 2). Membranes were then incubated for one hour with the HorseRadish Peroxidase (HRP) tagged antibody (1:5000 in PBS with 0.1% Tween). Chemiluminescence was obtained by using SuperSignal™ West Pico PLUS (Thermo Scientific ref 34,578) as HRP substrate and captured with the iBright™ CL1500 Imaging System (Thermo Scientific).

The integrated density of the specific band for each protein of interest was measured and normalized over the measure of the glyceraldehyde-3-phosphate dehydrogenase (GAPDH) protein. The results are reported as the ratio between the value of the protein of interest and the value of the corresponding control cells for each pool of cells (non-exposed cells) in arbitrary unit (AU). An example of each blot is seen in Supplementary Figs. 1 and 2.

### Data analysis

Image analysis from immunostaining or western blot was performed using the ImageJ software. The morphological analysis of the iRPE cells to obtain the cell area distribution was carried out using the images stained with Phalloidin and a macro developed on ImageJ by the CHIC (Centre d’Histologie, d’Imagerie et de Cytométrie, Centre de Recherche des Cordeliers UMR S 1138, Paris, France). Statistical analysis of the cell area distribution was done using a Chi-square test comparing the distribution of the exposed cells to the distribution of the non-exposed cells (n = 4 images per well, 2 wells per condition). For all other analysis, the non-parametric Kruskal–Wallis test (also called H test and which value is displayed in each figure) followed by Dunn’s post-tests were used to evaluate significant differences. Statistical analysis was performed using the GraphPad Prism 8.0.1 software. All graphs represent the median with the interquartile range.

### Supplementary Information

Supplementary Figure 2.

Supplementary Figure 1.

## Data Availability

The datasets generated during and/or analyzed during the current study are available from the corresponding author on reasonable request.

## References

[1] Duke-Elder WS (1926). The pathological action of light upon the eye. The Lancet.

[2] Favazza AR (1991). Literature on sun gazing. Am. J. Psychiatry.

[3] Noell WK, Walker VS, Kang BS, Berman S (1966). Retinal damage by light in rats. Invest. Ophthalmol..

[4] Ham WT, Mueller HA, Ruffolo JJ, Clarke AM (1979). Sensitivity of the retina to radiation damage as a function of wavelength. Photochem. Photobiol..

[5] Ham WT, Mueller HA, Sliney DH (1976). Retinal sensitivity to damage from short wavelength light. Nature.

[6] van Norren D, Gorgels TGMF (2011). The action spectrum of photochemical damage to the retina: A review of monochromatic threshold data. Photochem. Photobiol..

[7] Ham WT, Mueller HA, Ruffolo JJ, Guerry D, Guerry RK (1982). Action spectrum for retinal injury from near-ultraviolet radiation in the aphakic monkey. Am. J. Ophthalmol..

[8] Hunter JJ (2012). The susceptibility of the retina to photochemical damage from visible light. Prog. Retin. Eye Res..

[9] Jaadane I (2020). Retinal phototoxicity and the evaluation of the blue light hazard of a new solid-state lighting technology. Sci. Rep..

[10] Stevens AR (2023). Photobiomodulation in acute traumatic brain injury: A systematic review and meta-analysis. J. Neurotrauma.

[11] Geneva II (2016). Photobiomodulation for the treatment of retinal diseases: A review. Int. J. Ophthalmol..

[12] Canonica J (2021). Pathogenic effects of mineralocorticoid pathway activation in retinal pigment epithelium. Int. J. Mol. Sci..

[13] Jaadane I (2017). Effects of white light-emitting diode (LED) exposure on retinal pigment epithelium in vivo. J. Cell. Mol. Med..

[14] Bullough JD (2000). The blue-light hazard: A review. J. Illuminat. Eng. Soc..

[15] Ahn S-H, Suh J-S, Lim G-H, Kim T-J (2023). The potential effects of light irradiance in glaucoma and photobiomodulation therapy. Bioengineering.

[16] Françon A, Torriglia A (2023). Cell death mechanisms in retinal phototoxicity. J. Photochem. Photobiol..

[17] Belousova EA, Lavrik OI (2022). The role of PARP1 and PAR in ATP-independent nucleosome reorganisation during the DNA damage response. Genes.

[18] Kotoglou P (2009). Hsp70 translocates to the nuclei and nucleoli, binds to XRCC1 and PARP-1, and protects HeLa cells from single-strand DNA breaks. Cell Stress Chaperones.

[19] Kim YJ (2017). A protective mechanism of visible red light in normal human dermal fibroblasts: Enhancement of GADD45A-mediated DNA repair activity. J. Invest. Dermatol..

[20] Sergio LPS (2016). Low-intensity red and infrared lasers affect mRNA expression of DNA nucleotide excision repair in skin and muscle tissue. Lasers Med. Sci..

[21] Cuschieri J, Billigren J, Maier RV (2006). Endotoxin tolerance attenuates LPS-induced TLR4 mobilization to lipid rafts: A condition reversed by PKC activation. J. Leukocyte Biol..

[22] Aveleira CA, Lin C-M, Abcouwer SF, Ambrósio AF, Antonetti DA (2010). TNF-α signals through PKCζ/NF-κB to alter the tight junction complex and increase retinal endothelial cell permeability. Diabetes.

[23] Jaadane I (2015). The activation of the atypical PKC zeta in light-induced retinal degeneration and its involvement in L-DNase II control. J. Cell Mol. Med..

[24] Omri S (2013). PKCζ mediates breakdown of outer blood-retinal barriers in diabetic retinopathy. PLoS One.

[25] Zhang W, Liu H, Al-Shabrawey M, Caldwell RW, Caldwell RB (2011). Inflammation and diabetic retinal microvascular complications. J. Cardiovasc. Dis. Res..

[26] Chang S, Kim JH, Shin J (2002). p62 forms a ternary complex with PKCzeta and PAR-4 and antagonizes PAR-4-induced PKCzeta inhibition. FEBS Lett..

[27] Chen H (2006). Hsp70 inhibits lipopolysaccharide-induced NF-kappaB activation by interacting with TRAF6 and inhibiting its ubiquitination. FEBS Lett..

[28] Hamblin MR (2018). Mechanisms and mitochondrial redox signaling in photobiomodulation. Photochem. Photobiol..

[29] Evangelista AN (2021). Photobiomodulation therapy on expression of HSP70 protein and tissue repair in experimental acute Achilles tendinitis. Lasers Med. Sci..

[30] Intartaglia D, Giamundo G, Conte I (2022). Autophagy in the retinal pigment epithelium: A new vision and future challenges. The FEBS J..

[31] Dokladny K, Myers OB, Moseley PL (2015). Heat shock response and autophagy–cooperation and control. Autophagy.

[32] Khan I, Tang E, Arany P (2015). Molecular pathway of near-infrared laser phototoxicity involves ATF-4 orchestrated ER stress. Sci. Rep..

[33] Georgiou M (2022). Activation of autophagy reverses progressive and deleterious protein aggregation in PRPF31 patient-induced pluripotent stem cell-derived retinal pigment epithelium cells. Clinical and Translational Medicine.

[34] Glick D, Barth S, Macleod KF (2010). Autophagy: cellular and molecular mechanisms. J Pathol.

[35] Ploumi C, Papandreou M-E, Tavernarakis N (2022). The complex interplay between autophagy and cell death pathways. Biochem. J..

[36] Zhang T-Z (2014). Suppressing autophagy protects photoreceptor cells from light-induced injury. Biochem. Biophys. Res. Commun..

[37] Morishita H (2022). Role of autophagy in the eye: From physiology to disease. Curr. Opin. Physiol..

[38] Giansanti V (2013). Characterization of stress response in human retinal epithelial cells. J. Cell Mol. Med..

[39] Zatloukal K (2002). p62 is a common component of cytoplasmic inclusions in protein aggregation diseases. Am. J. Pathol..

[40] Hayden MS, Ghosh S (2008). Shared principles in NF-κB signaling. Cell.

[41] Kucharczak J, Simmons MJ, Fan Y, Gélinas C (2003). To be, or not to be: NF-κB is the answer – role of Rel/NF-κB in the regulation of apoptosis. Oncogene.

[42] Samuel W (2017). Appropriately differentiated ARPE-19 cells regain phenotype and gene expression profiles similar to those of native RPE cells. Mol. Vis..

[43] Singh R (2013). Functional analysis of serially expanded human iPS cell-derived RPE cultures. Invest. Ophthalmol. Vis. Sci..

[44] Udry F (2020). Lentiviral mediated RPE65 gene transfer in healthy hiPSCs-derived retinal pigment epithelial cells markedly increased RPE65 mRNA, but modestly protein level. Sci. Rep..

