# Supplementary figures and images for: Phototoxicity of low doses of light and influence of the spectral composition on human RPE cells
Source: Sci Rep. 2024 Mar 21;14:6839. doi: 10.1038/s41598-024-56980-9 (PMC10957882; doi:10.1038/s41598-024-56980-9)

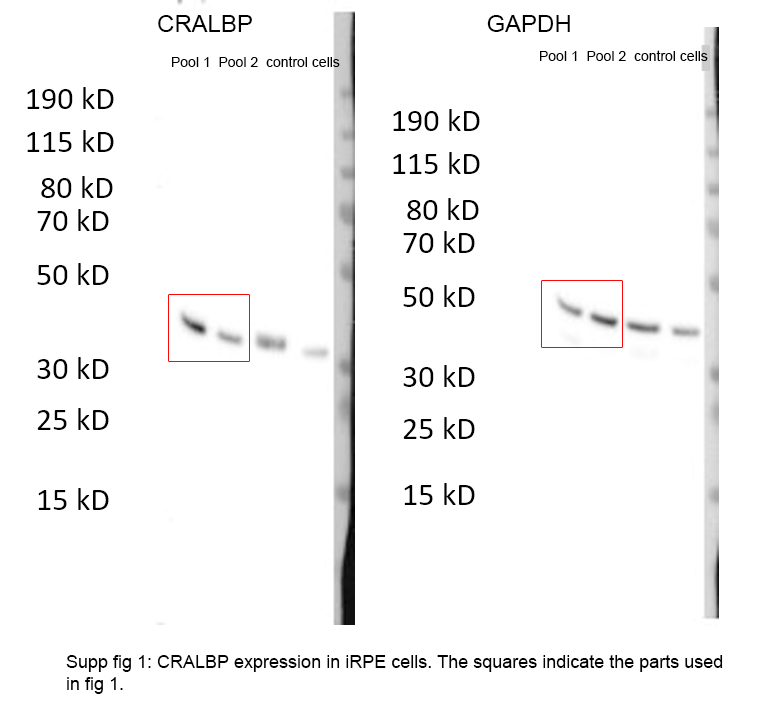

Supplement: Supplementary file 2 — Supplementary Figure 1. [file 41598_2024_56980_MOESM2_ESM.tif]
